# Ibero-American Consensus for the Management of Peritoneal Sarcomatosis: Updated Review and Clinical Recommendations

**DOI:** 10.3390/cancers16152646

**Published:** 2024-07-25

**Authors:** Francisco Cristóbal Muñoz-Casares, Javier Martín-Broto, Pedro Cascales-Campos, Juan Torres-Melero, Irene López-Rojo, José Gómez-Barbadillo, Luis González-Bayón, Ana Sebio, César Serrano, Sara Carvalhal, Joaquim Abreu de Souza, Alexandre Souza, Guillermo Flores-Ayala, Luis José Palacios Fuenmayor, Raquel Lopes-Bras, José Antonio González-López, Hugo Vasques, José Manuel Asencio-Pascual

**Affiliations:** 1 Peritoneal Carcinomatosis and Retroperitoneal Sarcomas Unit, San Juan de Dios Hospital, 14012 Córdoba, Spain; 2 Medical Oncology Department, Jimenez Diaz Foundation University Hospital, 28040 Madrid, Spain; 3 Peritoneal and Sarcomas Oncology Surgery Unit, Virgen de la Arrixaca University Hospital, 30120 Murcia, Spain; 4 Peritoneal Oncology Surgery Unit, Torrecárdenas University Hospital, 04009 Almeria, Spain; 5 Surgery Department, MD Anderson Cancer Center Madrid, 28033 Madrid, Spain; 6 Peritoneal Carcinomatosis Unit, Gregorio Marañón University Hospital, 28007 Madrid, Spain; 7 Medical Oncology Department, Santa Creu i Sant Pau University Hospital, 08025 Barcelona, Spain; 8 Medical Oncology Department, Vall D’Hebron University Hospital, 08035 Barcelona, Spain; 9 Surgery Department, Portuguese Institute of Oncology of Lisbon, 1099-023 Lisbon, Portugal; 10 Surgery Department, Portuguese Institute of Oncology of Porto, 4200-072 Porto, Portugal; 11 Surgery Department, Jalisco Institute of Cancerology, Guadalajara 44200, Mexico; 12 Surgery Department, Institute of Cancerology (IDC) Las Américas, Medellín 050025, Colombia; 13 Medical Oncology Department, Santa María Hospital, Lisboa North University Center, 1649-028 Lisboa, Portugal; 14 Surgery Department, Santa Creu i Sant Pau University Hospital, 08025 Barcelona, Spain; 15 Hepatobiliary Surgery and Liver Transplant Unit, Gregorio Marañón University Hospital, 28007 Madrid, Spain

**Keywords:** peritoneal sarcomatosis, soft tissue sarcoma, peritoneal GIST, peritonectomy procedures, cytoreductive surgery, hyperthermic intraperitoneal chemotherapy, induction chemotherapy, targeted therapy

## Abstract

**Simple Summary:**

Peritoneal sarcomatosis has traditionally been characterized by a poor prognosis and few effective treatment options. Improving radical surgery procedures with high percentages of complete cytoreduction and limited morbidity in centers with considerable experience, together with new systemic treatments associated with evolutionary development in oncology, may modify this concept. The wide histological variability of soft tissue sarcomas that can cause peritoneal sarcomatosis prompts its evaluation and treatment by expert multidisciplinary teams. An updated review of the scientific evidence in this regard and the consensus regarding recommendations for the adequate management of this condition represent the main objective of this study.

**Abstract:**

Peritoneal sarcomatosis is a rare malignant disease with a poor prognosis, secondary to peritoneal dissemination of abdominopelvic soft tissue sarcomas. Its rarity, together with the characteristic histological heterogeneity and the historically poor response to systemic treatments, has prevented the establishment of widely accepted treatment criteria with curative intent. In this sense, radical cytoreductive surgery (CRS) with peritonectomy procedures and hyperthermic intraperitoneal chemotherapy (HIPEC), widely used in peritoneal carcinomatosis with excellent results, have not had the same evolutionary development in patients with peritoneal sarcomatosis. A multidisciplinary working group of experts in sarcomas and peritoneal oncological surgery established a series of recommendations based on current scientific evidence for the management of peritoneal sarcomatosis, taking into account the different histological subgroups of abdominopelvic sarcomas that can cause it depending on their origin: retroperitoneal sarcomas, uterine sarcomas, and visceral/peritoneal sarcomas of GIST (gastrointestinal stromal tumor) and non-GIST origin. This article shows the results of sarcoma experts’ voting on the recommendations presented during the I Ibero-American Consensus on the Management of Peritoneal Sarcomatosis, which took place during the recent celebration of the III Hispanic-Portuguese Meeting for Updates on the Treatment of Sarcomas.

## 1. Introduction

Peritoneal sarcomatosis is caused by the peritoneal metastatic spread of soft tissue sarcomas (STSs) located in the abdominopelvic region. It represents a rare, advanced malignant neoplastic entity with an ominous prognosis. In line with the numerous and different histological subtypes of STSs, of which more than 100 are currently defined [1], peritoneal sarcomatosis may originate in a large proportion of them. For this reason, it is characterized as a rare malignant neoplasm that is very heterogeneous and extremely difficult to study to gather strong scientific evidence [2].

Peritoneal sarcomatosis usually occurs after the post-treatment recurrence of the primary sarcoma. In this sense, the most frequent causal factors for peritoneal seeding may be the multifocality of the tumor, the presence of unremoved satellite nodules after primary surgery, a possible traumatic dissection through the tumor capsule, or spontaneous or intraoperative ruptures of the primary sarcoma during surgical dissection [3].

The most common sarcomas that cause peritoneal metastases are retroperitoneal soft tissue sarcomas (RPSs), pelvic sarcomas such as uterine leiomyosarcomas (uLMSs), gastrointestinal stromal tumors (GISTs), and desmoplastic abdominal small round cell tumors (DSRCTs) in young adults or adolescents [4]. By taking this and a classification based on the abdominal location of the sarcoma that causes it to be taken into account, we distinguished four groups for specific study: retroperitoneal sarcomas, uterine sarcomas, and visceral/peritoneal sarcomas of GIST and non-GIST origin. Although each group may have its own histological variability, there are also global characteristics that differentiate them and define them as a group. Thus, although anyone can develop peritoneal sarcomatosis, visceral sarcomas usually have a greater capacity to produce liver metastases, retroperitoneal sarcomas have a greater capacity for pulmonary metastasis, and uterine sarcomas have a greater tendency for peritoneal dissemination, mainly due to rupture or extension due to iatrogenesis [3].

On the other hand, if the therapeutic cornerstone for the curative treatment of STSs is surgery, it should also be available for selected patients with peritoneal sarcomatosis. Fortunately, one of the historical milestones for oncological surgery occurred at the end of the 20th century, when Paul Sugarbaker systematized surgical procedures that allowed a radical surgical approach to peritoneal malignancy, applicable to patients with peritoneal carcinomatosis and sarcomatosis [5]. The promising results of radical cytoreductive surgery (CRS) and hyperthermic intraperitoneal chemotherapy (HIPEC) for peritoneal disease due to ovarian cancer, colon cancer, peritoneal mesothelioma, and pseudomyxoma peritonei have stimulated their use in peritoneal sarcomatosis. However, peritoneal sarcomatosis has not had the same acceptance in terms of the benefits and application of these surgical procedures. Of the three basic principles that underlie its application (the selection of patients with locoregional disease, the performance of radical surgery using peritonectomy procedures to eliminate macroscopic disease, and, finally, a combination of intraperitoneal and systemic chemotherapy to eliminate residual microscopic disease and control the disease tumor) [6], those referring to chemotherapy have been questioned the most in terms of the benefit of these procedures in peritoneal sarcomatosis. However, several factors can modify this pessimistic attitude. The first is the correct individualization of patients based on their tumor histology. The second is the systematization of these procedures, performed in expert centers with high percentages of complete cytoreduction and little associated morbidity. Lastly, we have the evolutionary development of medical oncology at the genetic and molecular levels, with the emergence of targeted treatments that increase effectiveness against certain types of sarcomas.

In such circumstances, the purpose of this study was to scientifically evaluate the current situation and develop a consensus regarding the recommendations among international experts for the clinical management of these patients with peritoneal sarcomatosis. With this objective, the I Ibero-American Consensus for the Management of Metastatic Disease in Sarcomas was held in 2024, with a special focus on peritoneal sarcomatosis and its therapeutic options.

## 2. Methodology

### 2.1. Working Group

A group of 18 professionals—experts in the multidisciplinary treatment of metastatic sarcomas—from different cities and Latin American countries was formed. Among these were 14 oncological surgeons with extensive experience in the radical surgical treatment of sarcomas and peritoneal oncological surgery for sarcomatosis. Likewise, four medical oncologists with extensive experience and exclusive dedication to sarcomas also participated. The working group was divided into four peritoneal sarcomatosis-specific subgroups: uterine sarcomatosis, retroperitoneal sarcomatosis, GIST visceral/peritoneal sarcomatosis, and non-GIST peritoneal/visceral sarcomatosis. A medical oncologist with proven experience in the field was included in each of the four subgroups.

One of the participating professionals led the working group, coordinated the four subgroups, and managed the organization and development of the topics to be discussed. He also collected all of the information generated in the documents written by the four subgroups in order to express it in an audiovisual presentation and discuss it on the day of the consensus meeting. Two other participants were dedicated to the general coordination of the international meeting and the final review of the documentation. During the 3-month working period, three online meetings were held with all members of the working group attending, except for the general coordinators, and three other specific meetings between the group coordinator and the general coordinators were also held.

### 2.2. Bibliographic Search

The working group conducted an in-depth search of the scientific publications cited in the MEDLINE database up to May 2024 on peritoneal sarcomatosis and radical cytoreductive surgery with peritonectomy and HIPEC procedures, using the PubMed database without limiting the publication date or other filters. The search was initially carried out using general terms such as “peritoneal sarcomatosis”, “peritoneal sarcomatosis and HIPEC”, “soft tissue sarcoma and HIPEC”, and “cytoreductive surgery and peritoneal sarcomatosis”. Subsequently, a selective search was carried out for each subgroup, with terms such as “uterine sarcomatosis”, “retroperitoneal sarcomatosis”, “peritoneal sarcomatosis and GIST”, and “GIST and HIPEC”. After any duplications were eliminated, the remaining publications of cytoreductive surgery and HIPEC for peritoneal sarcomatosis were selected, excluding those without peritoneal dissemination or without the administration of HIPEC.

Each of the four subgroups made a report of the available evidence and prepared a table with the most relevant published studies. Similarly, each subgroup consulted the clinical guidelines or review articles (for levels of evidence) that were published during the last 6 years in the PubMed database on the systemic treatment of the type of metastatic sarcomas of their own subgroup. The chosen studies were those published by the Spanish Sarcoma Research Group (GEIS), Clinical Practice Guidelines of the European Society of Medical Oncology (ESMO), European Reference Network for Rare Solid Cancers in Adults (EURACAN), European Reference Network for Genetic Tumor Risk Syndromes (GENTURIS), and Trans-Atlantic Retroperitoneal Sarcoma Working Group (TARPSWG).

With all of this information, 33 clinical recommendations were prepared and selected for their levels of evidence regarding the adequate management of peritoneal sarcomatosis, which would be voted on at the consensus meeting. The panel adopted levels of evidence (I to V) and grades of recommendation (A to C), adapted from those published by the Infectious Disease Society of America [7]. The coordinator of the working group and the two general coordinators re-evaluated and defined the final recommendations to present at the consensus meeting (Table 1).

### 2.3. Consensus Meeting

During the recent celebration of the III Spanish–Portuguese Update Meeting on the Treatment of Sarcomas in May 2024, the I Ibero-American Consensus on the Management of Peritoneal Sarcomatosis took place, in which the definitive recommendations of the working group were presented to vote upon. These recommendations were voted for by multidisciplinary experts in sarcomas, who were registered for this purpose. Voting could be carried out using the participants’ own telephone, using QR code capture and the Slido interactive platform.

Consensus was defined when the affirmative vote reached or exceeded 80% of the votes cast. A unanimous consensus was considered if it was 100%, a strong consensus was considered between 90 and 99%, and a weak consensus was considered between 80 and 89%. Less than 80% was defined as a lack of consensus.

## 3. Scientific Evidence of CRS-HIPEC Procedures in Peritoneal Sarcomatosis

Sugarbaker’s early work on CRS-HIPEC, showing his experience and systematization of radical surgical procedures with a complete cytoreductive objective, always included sarcomatosis as an important beneficiary [8]. In 1999, he published the first results of CRS using perioperative intraperitoneal chemotherapy, i.e., doxorubicin or doxorubicin plus cisplatin, in 30 patients with peritoneal sarcomatosis out of a total of 43 patients with recurrences of abdominopelvic sarcomas in the period 1989–1996. The degree of cytoreduction, the greater or lesser extension of the disease in the different abdominal compartments, and a PCI (peritoneal cancer index) of less or more than 13 all had an impact on survival [9].

A few years later, in 2005, Bonvalot et al. published the only randomized clinical trial to date, including 38 patients with peritoneal sarcomatosis (originating in retroperitoneal and visceral sarcomas) undergoing CRS and perioperative intraperitoneal chemotherapy, although they did not use HIPEC [10]. The comparison was between 19 patients with CRS versus 19 patients with CRS plus EPIC (early postoperative intraperitoneal chemotherapy). The survival results were excellent at around 40% at 5 years, based on surgery but without the influence of EPIC. This article could have been decisive in reducing the expectations generated by these procedures, along with other factors, such as the poor chemosensitivity that has historically been attributed to STSs. In this sense, the extensive development of studies with better scientific evidence for peritoneal carcinomatosis compared to the limited development in peritoneal sarcomatosis is significant. Thus, in ovarian peritoneal carcinomatosis, the maximum evidence of an improvement in the results has been accompanied by induction chemotherapy prior to CRS-HIPEC [11,12,13].

In relation to this, as shown in Table 2, only nine studies have been published that included more than 20 patients with peritoneal sarcomatosis and operated on using CRS and HIPEC procedures [9,14,15,16,17,18,19,20,21]. Studies with a small number of patients, mostly retrospective, with great histological heterogeneity and low level of evidence. However, the high percentages of complete cytoreduction, with little associated morbidity and evident improvement in survival results in centers with extensive experience in recent years, may be decisive for its reactivation.

In accordance with what has been mentioned, only two systematic reviews have been published. The first was published in 2011 by Munene et al. [22], which included nine studies, of which only five included HIPEC, with a total of 240 patients and very variable data for survival (overall range of 7–65% at 5 years), morbidity (9–44%), and postoperative mortality (0–11%), meaning it is only recommended for use in centers with significant experience. The second was the meta-analysis published in 2022 by Wong et al. [23]. This review included 16 studies with 320 patients of variable histology (retroperitoneal, visceral GIST, visceral non-GIST, and uterine) with CC0 results (no macroscopic residual disease) of 79%, a 5-year overall survival of 35%, and severe morbidity of 17%, with an average stay of 16 days. The conclusions of the meta-analysis were more positive for CRS-HIPEC than in the Munene study, with improved results in the selected patients, especially in patients with CC0 and low tumor burden.

In short, the increase in the percentage of complete cytoreduction associated with low morbidity and low PCI is key to the results. However, the wide variability of STSs makes a more specific study focused on its histological origin advisable.

### 3.1. Uterine Peritoneal Sarcomatosis: Evidence for CRS-HIPEC and Systemic Treatment

Uterine sarcomas are rare and represent fewer than 10% of malignant tumors of the uterus [24]. Their behavior is usually aggressive, and these patients have a worse prognosis than those with endometrial cancer. According to the latest WHO (World Health Organization) classification, the main types of uterine sarcomas are smooth muscle tumors, such as leiomyosarcoma (uLMS); endometrial stromal sarcomas (EESs), divided into high-grade (HG-EES) and low-grade (LG-EES); and undifferentiated sarcomas (UUSs) [25]. Before 2014, UUSs were included in the same group as HG-EESs, which limits the evaluation of older studies. Other types of uterine sarcomas are mixed epithelial and mesenchymal tumors, highlighting the adenosarcoma and a miscellaneous group that includes inflammatory myofibroblastic tumors, PEComa, or uterine tumors that resemble ovarian sex cord tumors (UTROSCTs) [26]. Carcinosarcoma is derived from a monoclonal neoplastic cell and has more of the characteristics of epithelial neoplasia than stromal neoplasia, which is why it was excluded by the WHO from the group of sarcomas in 2020.

uLMS is usually diagnosed postoperatively in presumably benign lesions and is associated with a high risk of recurrence and mortality [27]. uLMSs represent up to 80% of uterine sarcomas if we exclude carcinosarcomas or mixed mesodermal tumors. Most uLMS are high grade and have a 50–70% chance of relapse, which can be distant, local, or both [28]. In its management, two factors related to a worse prognosis due to the risk of recurrence must be taken into account: positive peritoneal cytology and the morcellation of apparently benign lesions with a subsequent diagnosis of malignancy that favors the development of peritoneal sarcomatosis [29,30,31].

EESs represent the second most common type of uterine sarcoma (20%) and are divided into high and low grades with very different natural histories [24,32]. LG-EESs are characterized by the presence of estrogen and progesterone receptors that will determine the therapeutic strategy. At the molecular level, there is a characteristic translocation that is not present in high-grade versions, which involves the short arm of chromosome 7 and the long arm of chromosome 17 with the production of the fusion protein of the JAZF1/JJAZ1 gene, which is present in 50% of patients [33]. LG-EESs have a good long-term prognosis. However, recurrences are high, especially peritoneal types, and may be late [34]. HG-EESs and UUSs have more aggressive behavior. HG-EESs also have a specific translocation (mainly YWHAE-NUTM2B or ZC3H7B-BCOR), which can help in their diagnosis. They do not normally express hormone receptors. Peritoneal and distant recurrences in HG-EESs and UUSs are more frequent and earlier after primary diagnosis [24].

Most studies of uterine peritoneal sarcomatosis focus mainly on uLMS and EESs since they represent the majority of cases. The use of CRS-HIPEC is not part of the usual clinical practice for this type of peritoneal sarcomatosis. A selection of the most representative articles regarding the experience of this radical cytoreductive treatment with the administration of HIPEC in patients with uterine sarcomatosis is reflected in Table 3 [14,16,18,21,35,36,37].

Most publications have used a combination of cisplatin, doxorubicin, and melphalan and include a limited number of patients in retrospective and heterogeneous studies, with the frequent inclusion of different histologies in the overall results, including non-uterine sarcomas. In fact, only three studies exclusively include patients with uterine peritoneal sarcomatosis [18,35,36]. However, a global view of the studies reflected in Table 3 allows us to observe some details to take into account. In the paper published by Díaz-Montes et al. [36], only 27% received treatment with CRS-HIPEC, obtaining better results compared to CRS alone. On the other hand, the results of Kusamura et al. [35], in 10 patients with a majority of uLMS and an overall survival of 65% at 5 years without morbidity and mortality associated with the procedure, are very interesting data to consider. The study conducted by Baratti et al. [16] and the multi-institutional study carried out by Sardi et al. [18] also present uLMS as a possible candidate to benefit from this surgical approach. Finally, the study recently published by Muñoz-Casares et al. [21] is not specific to uterine origin, but it individualizes the survival results of 10 patients with uterine peritoneal sarcomatosis, and it is the study with the largest number of EESs. In this last study, with global results of cytoreduction and morbidity and mortality similar to Kusamura et al. [35], LG-EES and uLMS are the sarcomatosis types that benefited the most from this surgical approach, compared to the poor results for HG-EES and UUS.

Although the evaluation of the studies in Table 3 does not allow us to draw conclusions with an adequate level of evidence to recommend the application of HIPEC, we can observe that certain histological types could benefit more from a cytoreductive surgical effort. Improving these results involves agreeing on the appropriate timing of the surgical intervention and the possible systemic induction treatment, which is always associated with knowledge of the specific histological type and its possible therapeutic options. In this sense, in Table 4, the key recommendations of GEIS in its Clinical Practice Guidelines are presented in relation to advanced or metastatic uterine sarcoma, recently published by Pérez-Fidalgo et al. [26].

### 3.2. Peritoneal Sarcomatosis of Retroperitoneal Origin: Evidence from CRS-HIPEC and Systemic Treatment

Among STSs, 15% are located in the retroperitoneum (RPSs). RPSs are rare diseases that represent 0.15% of all malignant tumors in adults. This tumor’s retroperitoneal location, without internal structures that limit its growth, predisposes it to its large size and gives it the characteristic of being extraordinarily difficult to remove. Moreover, even after the complete resection of these tumors, recurrence is very common [38]. This is likely a consequence of the challenging nature of RPS surgery, but is also partly due to the biology of the disease itself. Different RPS histologies are characterized by diverse biological behaviors with different early and late risks of local and distant recurrence. Thus, peritoneal metastases from primary RPS are rare and most of them occur as a progression or locoregional recurrence of the same tumor, especially when the primary tumor has ruptured spontaneously or surgically [3].

In the modern era of precision medicine, our understanding of the implications of tumor subtype biology and anatomical location has led to a more nuanced and specific therapeutic approach based on tumor histology [39]. In this sense, taking into account that the liposarcomas of the retroperitoneum comprise more than 50% of RPSs and that their association with leiomyosarcomas reaches 80%, it is necessary to consider the most frequent subtypes: dedifferentiated liposarcoma (DDLPS), well-differentiated liposarcoma (WDLPS), leiomyosarcoma (LMS), solitary fibrous tumor (SFT), malignant peripheral nerve sheath tumor (MPNST), and undifferentiated pleomorphic sarcoma (UPS).

The most frequent of all is DDLPS, representing approximately 35% of RPSs. They can be intermediate- (grade 2) or high-grade (grade 3) tumors. The first group has a high tendency for local relapse (about 44% at 5 years), but a low risk of distant metastasis (about 10% during the same period). High-grade DDLPSs, on the other hand, have a similar or slightly lower risk of local relapse (33% at 5 years), but a significantly higher risk of distant metastasis (around 44% at 5 years) [40,41].

The next most frequent, with 25% of the RPSs, is the WDLPSs. They are low-grade tumors that commonly reach large dimensions before becoming symptomatic and being diagnosed. The survival of patients with WDLPSs is much higher than that of DDLPSs (87% at 5 years compared to 54% in those with intermediate-grade tumors and 41% in those with high-grade tumors). They have a limited potential for early local recurrence, but require long-term follow-up due to their possibility of recurring decades after primary resection. At 5 years, local recurrence can reach 30% [40,41].

LMSs represent approximately 20% of RPSs. Most commonly, they arise from the major abdominal veins, particularly the inferior vena cava, renal veins, and gonadal veins. LMSs are characterized by a high risk of distant metastasis and a low risk of local relapse. In fact, about 50–60% of LMS patients have metastases in the first 5 years, while less than 10% will recur locally in the same time period. In this histology, the prognosis will depend more on hematogenous dissemination than on local recurrence [40,41].

SFTs, occurring much less frequently than the previously mentioned sarcomas and also being less involved in the development of peritoneal sarcomatosis, represent approximately 6% of all RPS histologies. Around 10% of them exhibit aggressive behavior and harbor metastatic potential. The local recurrence rate of retroperitoneal SFT is low compared to that of other histologies (around 5% at 5 years) and, together with WDLPSs, they are the retroperitoneal STSs with the best survival after their primary diagnosis (around 85% at 5 years) [40,41].

On the other hand, both MPNST and UPS each separately represent 3% of all RPS histologies. MPNST is characterized by a significant risk of local relapse and distant metastasis (15–20%) and may occur in patients with neurofibromatosis. As for UPS, it has a high rate of local recurrence and distant metastasis, generally in the lungs, which gives it a very poor prognosis [40,41].

Complete initial resection will not prevent more than 50% of patients treated for RPSs from relapse, and approximately 20–25% will develop distant metastases. In the case of peritoneal metastatic dissemination, the prognosis is very poor, given the primary location of these sarcomas and the extreme difficulty in achieving complete cytoreduction along with the tendency toward continuous recurrence. However, the wide histological variability of these tumors makes it advisable to consider them individually and evaluate them using an expert multidisciplinary team [21,42]. For three decades, Sugarbaker’s procedures have guaranteed the possibility of greater surgical radicalness in certain selected patients with peritoneal malignancy. Thus, Table 5 shows the main published series based on the results of patients with peritoneal sarcomatosis undergoing surgery using CRS and HIPEC [9,14,16,19,20,43,44,45,46].

The evaluation of the most representative series, presented in Table 5, shows that the majority of studies are retrospective, with a small number of patients and a great diversity of histological subtypes included. The most involved type of RPS, due to its characteristics and frequency, is the LPS, followed by the LMS. The most commonly used cytostatics as a HIPEC were doxorubicin, cisplatin, and mitomycin C. The study that provides the most patients with 34 peritoneal sarcomatosis of retroperitoneal origin is the prospective multicenter study conducted by Rossi et al. [14], with overall 5-year survival figures of around 40%, similar to the series by Randle [43] and Karamveri et al. [46]. On the other hand, it is interesting to note the intentionality of the study by Almasri et al. [20], combining intravenous and intraperitoneal hyperthermic chemotherapy intraoperatively after performing surgical cytoreduction. The absence of postoperative mortality and the 2-year survival results shown in this series of patients are a stimulus to develop multidisciplinary studies that allow for expanding scientific knowledge and therapeutic options in this field.

In this sense, knowing the current evidence on the best systemic treatments for this type of peritoneal sarcomatosis can allow the multidisciplinary team to consider induction or perioperative treatments that optimize surgical efforts and patient results. Table 6 summarizes some clinical recommendations in advanced or metastatic RPSs, such as those published by Álvarez et al. from GEIS [47] in accordance with those of ESMO-EURACAN-GENTURIS [48] and those of the TARPSWG [42,49].

### 3.3. Peritoneal Sarcomatosis Of Origin In GIST Visceral/Peritoneal Sarcomas: Evidence In CRS-HIPEC And Systemic Treatment

Gastrointestinal stromal tumors (GISTs) are the most common neoplasms of mesenchymal origin from the digestive tract. They originate from the stromal Cajal cells. Their most frequent location is the stomach (60%), followed by the jejunum and ileum (30%), and they may appear in other, less frequent locations such as the duodenum, colon, rectum, or esophagus. Metastases are present at diagnosis in approximately 15% of patients and are typically found in the peritoneum and liver. Bone and lung metastases are rare and rarely metastasize through the lymphatic system [50,51].

One of the main characteristics of GISTs is that they present alterations in two tyrosine kinase receptors encoded by the KIT and PDGRFA genes, targets against which targeted therapies have been developed, radically changing the prognosis of these tumors. As a result, five tyrosine kinase inhibitors (TKIs) currently have regulatory approval for the treatment of metastatic GIST: imatinib, sunitinib, regorafenib, ripretinib, and avapritinib [52].

In such circumstances, the determination of the molecular profile of GISTs is essential to adapt the use of specific agents approved against this disease. Thus, approximately 85% of GISTs are driven by mutually exclusive activating mutations in KIT or PDGFRA. However, 10-15% of GISTs are wild-type (WT) for KIT and PDGFRA, and other events are involved in their pathogenesis [53].

With the introduction of imatinib in 2002, previous 5-year overall survival rates of less than 10% improved significantly with acceptable systemic toxicity [54]. For this reason, the initial treatment of advanced GIST is based on TKI (mainly imatinib). However, imatinib is not a curative treatment and should be associated with cytoreductive surgery to achieve better long-term results. In this sense, despite the excellent initial results with TKIs (80% initial response), many patients develop resistance to treatment and die after the progression of their disease [55]. Thus, although complete radiological responses have been described in around 2% of patients with metastatic GISTs treated with imatinib [56], it remains anecdotal. Around 50% of metastatic or progression patients treated with imatinib develop resistance to treatment in the first two years, with 10% of these resistances occurring during the first 6 months [57]. Progression-free survival due to the development of resistance is significantly lower for sunitinib (5.8 months), regorafenib (4.8 months), and ripretinib (6 months) [56]. Primary resistance to imatinib is related to WT-KIT tumors or mutations other than exon 11. However, the mechanism of resistance to treatment or pressure from TKIs is usually the acquisition of additional mutations in the KIT gene, which is different from primary resistance [53,58].

The combination of neoadjuvant TKI and surgical cytoreduction in selected patients achieves better results than treatment with TKI alone [58,59,60]. This attitude is also recommended in the case of peritoneal dissemination, called peritoneal GISTosis, which is the second most common form of GIST recurrence after hepatic. Peritoneal recurrence is usually related to spontaneous or iatrogenic rupture during the surgical procedure for primary GIST. This tumor rupture, described in around 9% of GIST surgeries [61], is a well-established risk factor for peritoneal recurrence. In these circumstances of peritoneal GISTosis, cytoreduction with peritonectomy procedures represents the type of surgery with the best guarantees to achieve complete cytoreduction. However, based on the poor activity of conventional chemotherapy used intraperitoneally (doxorubicin, cisplatin, and mitomycin C) against GIST, the administration of HIPEC is a controversial issue and is not accepted with the same rational basis as in other malignant diseases of the peritoneal surface.

Table 7 shows the few published studies of series with peritoneal GISTosis treated with CRS-HIPEC [14,15,16,21,62,63]. The initial evaluation of this table allows some comments on it. In this sense, the first studies included patients with GIST from the pre-imatinib era along with other non-GIST sarcomatosis; there were modest results in the two studies, in which the results of patients with GIST [16,62] were individualized and reflected. On the other hand, the most representative study (because it is specific to peritoneal GISTosis, with the largest number of patients) is that published by Bryan et al. [63]. This study analyzes a period that includes the years 1992–2012, with 16 patients, of whom 50% were treated during the pre-imatinib era. In this study, the importance of TKI resistance was analyzed. Those patients with a response to imatinib had excellent results compared to the disappointing results in patients with progression, even if they had achieved complete cytoreduction. Surprisingly, the next study published on these radical surgical procedures with HIPEC, ten years later, is the one by Muñoz Casares et al. [21]. This study analyzes a period between 2016 and 2022, which is in the midst of the imatinib era. The survival results in patients with peritoneal GISTosis were particularly encouraging, with a 5-year overall survival (OS) of 80% and a 5-year disease-free survival (DFS) of 33%. In this study, all patients underwent surgery after showing a response to TKI.

Recurrences due to resistance to prolonged treatment with TKI, such as imatinib, could allow for the development of a new radical surgical approach after achieving a new response with another TKI [50] and avoiding surgery for peritoneal GISTosis in progression that has not shown benefit [64], except in situations with a global response and oligoprogression that could be resectable [65].

It is evident that in the case of peritoneal GISTosis, the decision of the expert multidisciplinary team acquires very important significance based on the need to achieve a response and stop the progression before evaluating the possibility of radical surgery in selected patients. In relation to this, in Table 8, we show the current recommendations regarding the treatment of metastatic GIST, published in the recent GEIS Clinical Practice Guidelines by Serrano et al. [53], and those of ESMO-EURACAN-GENTURIS by Casali et al. [66].

### 3.4. Peritoneal Sarcomatosis Of Origin In Non-GIST Visceral/Peritoneal Sarcomas: Evidence in CRS-HIPEC and Systemic Treatment

Various studies focused on the differential diagnosis of the different neoplastic pathologies that affect the peritoneum, such as the works of Miguez-González, Oei, and Pickhardt et al. [67,68,69], correlated with the search for the different etiologies of peritoneal sarcomatosis shown in the medical literature, have allowed us to recognize the main histological origins involved in this subgroup.

Thus, due to its frequency and tendency to be located in the peritoneum compared to others that are more common in the retroperitoneal space (LPS, LMS, SFT, MPNST, and UPS) and that were treated in their corresponding section, these other three deserve special attention: the desmoplastic small round cell tumor (DSRCT), rhabdomyosarcoma (RMS), and epithelioid inflammatory myofibroblastic sarcoma (EIMS).

#### 3.4.1. Desmoplastic Small Round Cell Tumor (DSRCT)

DSCRT is a very rare and aggressive soft tissue sarcoma that primarily affects adolescent and young adult males. It arises mainly in the abdomen and presents peritoneal sarcomatosis in more than 90% of cases at the time of diagnosis and around 50% of synchronous extraperitoneal metastases, mainly located in the lymph nodes, liver, and lungs. It is characterized by a chromosomal translocation t(11:22) (p13;q12), which leads to the fusion of the Ewing sarcoma gene (ESW1) with the Wilms tumor gene (WT1) [70,71].

Despite its notable chemosensitivity, the prognosis of DSRCT is dismal, with a 5-year overall survival rate of 10–25% [72,73]. Currently, the best available treatment should combine induction chemotherapy with complete CRS. The most effective chemotherapy regimen is under debate, although most combinations are based on alkylating agents similar to that for Ewing’s sarcomas [73,74,75]. The response to systemic chemotherapy will be an indicator of the biology of the tumor that will help, together with the tumor burden (PCI) and the assessment of possible extra-abdominal disease, to make the most appropriate surgical decision [74,76,77]. Tumor progression after neoadjuvant chemotherapy alerts us to tumor biology that does not benefit from subsequent surgery [74]. The administration of HIPEC, with the intention of improving the postoperative microscopic cytoreduction that we can achieve with aggressive surgery that includes peritonectomy procedures, is a therapeutic modality frequently used in this type of peritoneal sarcomatosis, with Hayes-Jordan as its main representative [17,77]. Table 9 includes the most representative publications of this type of non-GIST peritoneal sarcomatosis of visceral origin, in which HIPEC was administered after CRS [2,17,77,78,79,80,81,82].

Table 10 summarizes the current evidence of the systemic treatment of advanced DSRCT disease that will allow us to establish the best therapeutic strategy against peritoneal sarcomatosis of this origin, including the GEIS recommendations published by Martinez-Trufero et al. [72,73,74,75].

#### 3.4.2. Rhabdomyosarcoma (RMS)

RMS is a high-grade soft tissue sarcoma with the phenotypic and biological characteristics of skeletal muscle cells. It includes four subtypes: embryonic (ERMS), which represents 80% of all RMSs, alveolar (ARMS), pleomorphic (PRMS), and spindle cell/sclerosing (SRMS). RMS accounts for approximately 50% of all soft tissue sarcomas in the pediatric population [83]. The abdomen is a rare location seen in 10–12% of patients, with primary peritoneal RMS being an extremely unusual entity.

RMS peritoneal disease usually occurs secondarily to tumor dissemination from a primary abdominal or retroperitoneal site, either spontaneously or via the manipulation of the tumor. The transfer to the pediatric age of radical surgical management of peritoneal malignant disease in adults, using CRS and HIPEC, has made it possible to perform this in patients with peritoneal sarcomatosis due to RMS. Thus, there are numerous publications on the matter, as shown in Table 9. However, the number of patients included in these studies is very small and does not exceed seven in any of them, as shown in the works of Hayes-Jordan et al. [17], Zhu et al. [82], and Geshe et al. [80]. Although we do not have studies that clarify the true importance of HIPEC, it has been possible to respond to the initial criticisms and convey the possibility of performing them with minimal morbidity in reference centers, even in early childhood [80].

Finally, in relation to the current scientific evidence of multidisciplinary treatment in advanced RMS, there are important messages to consider that are associated with different degrees of recommendation [83,84,85], as shown in Table 10.

#### 3.4.3. Epithelial Inflammatory Myofibroblastic Sarcoma (EIMS)

An inflammatory myofibroblastic tumor (IMT) is an extremely rare mesenchymal neoplasm composed of myofibroblastic spindle cells in a myxoid–collagenous stroma with an infiltration of inflammatory cells (mainly lymphocytes and plasma cells); in a third of cases, this is associated with a febrile inflammatory syndrome. It usually affects children and young adults, with the abdominopelvic location being the most common. However, it can occur at any age and in any area of the body, and is currently considered a mesenchymal neoplasm with intermediate biological potential [86,87].

A highly invasive variant of IMT called epithelioid inflammatory myofibroblastic sarcoma (EIMS) has recently been included in the 2020 World Health Organization Classification of Soft Tissue Tumors [88], as suggested by Mariño-Enriquez [89], with very aggressive behavior. Thus, in the largest published study of IMT in adults [87], which included 30 patients with a mean age of 38 years and a range of 21 to 77 years, 23% of patients had IMTs. Patients with this variant showed more aggressive tumors, all of them originating in the abdominal cavity and difficult to resect due to their large extension, and with 100% ALK positivity. In this study, the treatment of EIMS with crizotinib (first-line therapy directed against ALK) demonstrated a similar prognosis to the rest of the patients without EIMS. The review by Gros et al. [90], in relation to all publications on EIMS until 2022 and including 58 patients, confirmed the greater oncological aggressiveness of EIMS, its frequent location in the abdomen and pelvis, and the common presence of fusion mutations in ALK. Along these lines, a study of 12 children with EIMS, a rare variant of IMT in pediatric ages, has recently been published. Complete surgery, together with the multidisciplinary therapeutic combination that includes targeted therapies, improved the results of the study [91].

Given that surgery is the basis of treatment and that the intra-abdominal dissemination of these tumors is common, surgery with peritonectomy procedures and multivisceral resections (CRS) is required to achieve complete cytoreduction [92,93]. In this sense, the experience in this type of neoplasm of the combined treatment of CRS and intraperitoneal chemotherapy is very scarce and is limited to several publications of clinical cases. A 5-year-old child to whom HIPEC was administered and a 5-month-old infant to whom intraperitoneal chemotherapy was administered under conditions of normothermia showed good results and low morbidity in both cases [82,94].

In this type of peritoneal sarcomatosis, by taking into account the current evidence of systemic treatment, we can highlight certain important concepts and recommendations [95,96,97], as shown in Table 10.

**Table 10:** Clinical recommendations applicable in the systemic treatment of peritoneal sarcomatosis originating from non-GIST visceral sarcomas.

**Clinical recommendations and levels of evidence in the systemic treatment of advanced or metastatic soft tissue sarcomas (DSRCT, RMS, and EIMS) according to published Clinical Practice Guidelines [72,73,74,75,83,84,85,95,96,97]**
**Desmoplastic small round cell tumor (DSRCT)**
√ The standard of care for patients presenting without extra-abdominal metastases is multimodal therapy with multiagent intensive CT and aggressive debulking surgery (IV, B). √ Most combinations are based on alkylating agents, similar to those in Ewing sarcomas, and include combinations of doxorubicin, vincristine, dactinomycin, cyclophosphamide, ifosfamide, and etoposide (IV, A). √ Once cytoreduction is completed, the role of hyperthermic peritoneal perfusion with CT (HIPEC) using cisplatin, remains unclear (V, C). √ Topoisomerase-containing regimens such as temozolomide/irinotecan or cyclophosphamide/topotecan, high-dose ifosfamide, and gemcitabine/docetaxel, are common second- and third-line regimens in recurrent DSRCT (V, B). Irinotecan and trabectedin seem promising drugs, due to their multifaceted mechanisms of action, combined with other cytostatics or even among themselves. √ There are new therapeutic options under investigation. In this sense, we highlight several targeted treatments, especially with tyrosine kinase inhibitors such as sunitinib, imatinib, sorafenib, or pazopanib, with or without mTOR inhibitors, angiogenesis blocking therapies in combination with irinotecan and temozolamide, immunotherapies directed at specific surface treatments of DSRCT, as well as therapies for androgen receptor pathway inhibition or insulin growth factor inhibition (V, C).
**Rhabdomyosarcoma (RMS)**
√ Chemotherapy is used in both the neoadjuvant, adjuvant, and metastatic settings. For adult pleomorphic and spindle cell RMS, it is usually doxorubicin. Chemotherapy regimens for the other most common subtypes include ifosfamide, vincristine, actinomycin D, doxorubicin, cyclophosphamide, and vinorelbine (IV, A). √ Maintenance chemotherapy with vinorelbine and low-dose cyclophosphamide in patients with RMS has been shown to improve survival (II, A).
**Epithelioid inflammatory myofibroblastic sarcoma (EIMS)**
√ Complete surgery is the cornerstone of EIMS treatment, along with a multidisciplinary therapeutic combination that includes targeted therapies (IV, A). √ In patients with locally advanced or metastatic disease in whom complete surgery is not contemplated and who associate ALK mutations, targeted therapy with ALK inhibitors (crizotinib as the first option, then alectinib and ceritinib) is the standard first-line treatment (II, A). √ Conventional chemotherapy also appears to be active in this disease, with responses around 50% using regimens based on anthracyclines or methotrexate plus vinca alkaloids, regardless of ALK status (IV, A). Responses have also been observed with oral cyclophosphamide and docetaxel/gemcitabine, but few patients were treated with these regimens.

#### 3.4.4. Less Common STS-derived Peritoneal Sarcomatosis

The other STSs included in this subgroup that can develop peritoneal sarcomatosis much less frequently are the following:

*Disseminated peritoneal leiomyomatosis (DPL) with malignant degeneration*: DPL is a rare clinicopathological entity that is characterized by the presence of multiple leiomyomas on the peritoneal surface of the abdominal and pelvic cavity, resembling a diffuse peritoneal malignancy. It is typically observed in women of reproductive age, and the most frequently related etiopathogenic factors have been hormonal dependence in patients with positive estrogen and progesterone receptors, as well as iatrogenic origin, after myomectomy and laparoscopic morcellation [98]. However, we have decided to include this pathology in this subgroup because it can also affect postmenopausal women and male patients [99,100,101]. In this sense, the etiopathogenesis is not so clear, and with hormonal dependence being ruled out in these cases, there are other theories, such as the possible metaplastic potential of stem cells within the peritoneal cavity and the genetic theory that would justify some cases in family groups [102]. Although it is considered histologically benign, it has invasive and recurrent potential, with exceptional capacity for sarcomatous malignancy, and may trigger peritoneal sarcomatosis [100,101,103]. Although the histological diagnosis should guide us regarding treatment, complete cytoreductive surgery will continue to be the treatment of choice. Moreover, in this pathology, there are publications regarding the combined use of CRS with cisplatin-based HIPEC, specifically in a case of recurrent DPL associated with endometriosis, although the role of HIPEC is unknown [104].

*Fibrosarcoma:* This is a malignant fibroblastic tumor with variable collagen production, which presents as a high-grade tumor in 80% of cases, affecting the deep soft tissues of the extremities, trunk, head, and neck, and, occasionally, the visceral organs. These tumors have been included in some studies of sarcomatosis treated with CRS and HIPEC, although without an analysis of specific results [9,14,19,43]. Although the response rate to radiotherapy and chemotherapy is very low, they are widely used as the neoadjuvant and/or adjuvant treatment of tumors. In advanced disease, doxorubicin as the first-line treatment and trabectedin, pazopanib, and combinations with gemcitabine as second-line treatments, are the alternatives for palliation [95].

*Angiosarcoma*: These are aggressive tumors that arise from vascular cells and can develop in previously irradiated tissues. The most common locations are the skin (mainly the scalp) and breast, but a small group (<20%) have a primary visceral location (lung, liver, heart, and kidney). Local extension and metastasis are common. In metastatic disease, it is highly sensitive to taxanes, which may be a treatment option over anthracyclines in this histological type [48]. An alternative option is gemcitabine, either alone or in combination with docetaxel. CRS and HIPEC for peritoneal metastasis, in the absence of systemic disease, is a possible therapeutic option, although it requires investigation [95,105].

*Synovial sarcoma*: This is a malignant tumor of uncertain differentiation (despite the name, it does not originate in synovial cells). It can be located in any part of the body, although in 70% of cases, it is found in the deep soft tissues of the extremities. There are some cases of synovial sarcoma included in a global study of peritoneal sarcomatosis treated using CRS-HIPEC [44,62]. Metastatic synovial sarcoma shows better responses to chemotherapy compared to other sarcomas. The standard of chemotherapy is doxorubicin combined with ifosfamide, with a response rate of 40%. Neoadjuvant therapy may facilitate surgery. For second-line chemotherapy, ifosfamide is often chosen. Trabectedin is also another possibility. Immunotherapy or targeted therapies are other options under investigation [106].

*Clear cell sarcoma*: It is also known as melanoma of soft parts; it is highly aggressive and arises from the deep soft tissues of the extremities (preferably distal) in young adults and has been rarely shown in other locations such as the abdomen and, very exceptionally, involving peritoneal sarcomatosis [107,108]. There is nodal and distant involvement in 30% of cases. This sarcoma is considered an entity that is not very sensitive to classic cytotoxic agents. Antiangiogenic agents, such as anlotinib and pazopanib, are active in this entity and could be offered as first-line treatments if available. Crizotinib, a MET receptor tyrosine kinase inhibitor, showed moderate activity [75].

## 4. Clinical Recommendations and Consensus for the Management of Peritoneal Sarcomatosis

The clinical recommendations with strong evidence that were presented and voted on by an average of 43 multidisciplinary sarcoma experts in the I Ibero-American Consensus on the Management of Peritoneal Sarcomatosis are set out in Table 11. All recommendations obtained the consensus of the experts by exceeding 80% of the affirmative votes. Of these, unanimity of consensus was obtained in 21% of the voted recommendations, strong consensus in 58%, and weak consensus in 21%.

## 5. Conclusions

This review and consensus article presents peritoneal sarcomatosis as a very diverse and heterogeneous entity, far from a single global consideration, and inaccessible by any treatment with curative intent. Thus, current evidence shows that radical surgery with peritonectomy procedures achieves the best results in patients with low-grade peritoneal sarcomatosis, such as well-differentiated liposarcoma or low-grade endometrial stromal sarcoma. Worse outcomes are associated with high-grade, sarcomas, such as dedifferentiated liposarcomas, high-grade endometrial stromal sarcomas, and undifferentiated sarcomas. The same treatment for other tumors, such as uterine leiomyosarcomas and desmoplastic small round cell tumors, also show promising results. In this sense, the use of induction treatment and effective targeted therapies that are combined with complete cytoreductive surgeries, as in the case of peritoneal GISTosis, is achieving results that were not imaginable years ago.

Regarding the issue of HIPEC, we currently do not have evidence that allows us to standardize and recommend its use in any specific histological type of peritoneal sarcomatosis, except for its probable null influence on GIST with the generally used intraperitoneal cytostatics. However, it is necessary to continue investigating its more-than-probable contribution in certain histologies, as has occurred in peritoneal carcinomatosis.

In short, a multimodal approach to high-volume centers with expert multidisciplinary teams that identify the histological type responsible for sarcomatosis and allow for the interrelation of the best systemic and targeted treatments, together with the performance at the most opportune time of radical surgical procedures that achieve high percentages of complete cytoreduction with minimal morbidity, are the key objectives for improving both the quality of life and the survival results for these patients. A multidisciplinary consensus based on the best available evidence and the commitment to multi-institutional collaboration to develop future high-quality clinical studies may be the instruments that allow us to obtain the best results.

## Figures and Tables

**Table 1:** Levels of evidence (I to V) and grades of recommendation (A to C).

**Levels of Evidence**	
I	Evidence from at least one large, randomized, controlled trial of good methodological quality (low potential for bias), or meta-analyses of well-conducted randomized trials without heterogeneity
II	Small randomized trials or large randomized trials with a suspicion of bias (lower methodological quality), or meta-analyses of such trials or of trials with demonstrated heterogeneity
III	Prospective cohort studies
IV	Retrospective cohort studies or case-control studies
V	Studies without a control group, case reports, and experts’ opinions
**Grades of Recommendation**	
A	Strong evidence for efficacy with a substantial clinical benefit, strongly recommended
B	Strong or moderate evidence for efficacy but with a limited clinical benefit, generally recommended
C	Insufficient evidence for efficacy or benefit does not outweigh the risk or the disadvantages (adverse events, costs…), optional

**Table 2:** Published series of >20 patients with peritoneal sarcomatosis undergoing surgery using CRS-HIPEC.

AUTHOR	Berthet [9]	Rossi [14]	Lim [15]	Baratti [16]	Hayes-Jordan [17]	Sardi [18]	Spiliotis [19]	Almasri [20]	Muñoz-Casares [21]
**YEAR**	1999	2004	2007	2010	2015	2017	2021	2024	2024
**PATIENTS**	43	60	28	37	34	36	21	29	23
**TIME FRAME**	1989–1996	1997–2002	1998–2003	1996–2006	NR	2005–2014	2005–2019	2017–2021	2016–2022
**STUDY** **DESIGN**	Retrospective single-center	Prospective multicenter	Prospective Phase 1. Non-randomized	Retrospective single-center	Retrospective single-center	Retrospective multicenter	Retrospective multicenter	Retrospective single-center	Retrospective single-center
**PRIMARY** **TUMOR ** **HISTOLOGY**	22 LMS, 9 LPS, 4 FS, 4 DSRCT, 1 MPNST	14 GIST, 12 uterines (8 uLMS, 4 EES), 34 RPS (20 LPS, 6 UPS)	17 LMS/GIST, 5 DSRCT, 2 LPS, 4 others	13 LPS, 11 uLMS, 8 GIST pre-Imatinib, 5 others	21 DSRCT, 7 RMS, 2 LPS, 4 other sarcomas, 12 other tumors	9 uLMS, 3 EES, 3 AS	7 LPS, 6 LMS, 4 RMS, 4 FS	12 LPS, 7 LMS, 3 FS	10 uterines (5 EES, 3 uLMS, 2 UUS), 6 GIST, 5 visceral non-GIST, 2 LPS
**CCS**	CC0-1: 63%	CC0: 68% CC0-1: 100%	CC0-1: 95%	CC0: 76% CC0-1: 84%	CC0: 95% CC0-1: 100%	CC0-1: 94%	CC0: 52% CC0-1: 90%	CC0: 52% CC0-1: 69%	CC0: 87% CC0-1: 96%
**HIPEC**	HIPEC with cisplatin (3 HIPEC, 13 HIPEC + EPIC), 14 EPIC, 13 No	doxorubicin + cisplatin	19 HIPEC: cisplatin 9 HIPEC: cisplatin + mitoxantrone	doxorubicin + MMC or cisplatin	cisplatin	22 doxorubicin + cisplatin, 10 melphalan, 4 others: cisplatin/MMC	11 MMC, 7 doxorubicin, 3 cisplatin	ifosfamide iv + HIPEC (24 doxorubicin + cisplatin, 5 doxorubicin + MMC)	16 doxorubicin + cisplatin, 4 doxorubicin, 3 paclitaxel

Abbreviations: AS: angiosarcoma; CCS: completeness of cytoreduction score; DSRCT: desmoplastic small round cell tumor; EES: endometrial stromal sarcoma; EPIC: early postoperative intraperitoneal chemotherapy; FS: fibrosarcoma; GIST: gastrointestinal stromal tumor; HIPEC: hyperthermic intraperitoneal chemotherapy; LMS: leiomyosarcoma; LMS/GIST: undifferentiated origin in the absence of molecular study; LPS: liposarcoma; MMC: mitomycin C; MPNST: malignant peripheral nerve sheath tumor; NR: not referred; RMS: rhabdomyosarcoma; RPS: retroperitoneal sarcoma; uLMS: uterine leiomyosarcoma; UPS: undifferentiated pleomorphic sarcoma; UUS: undifferentiated uterine sarcoma.

**Table 3:** Published series of patients with uterine peritoneal sarcomatosis undergoing surgery using CRS-HIPEC.

Author and Year	Design Study	Patients (n)	Primary Tumor Histology	HIPEC	PCI	CCS (%)	Morbidity G 3,4 (%)	Mortality 30 d (%)	DFS-5y (%)	OS-5y (%)	OS Median (months)
**Rossi** 2004 [14]	Prospective (multicenter)	12 of 60	8 uLMS, 4 EES	doxo + cisplatin	mean 7.7 (2–21)	Overall CC0: 68 CC0-1: 100	Overall 23	0	ND	Overall 38	ND Overall 34
**Kusamura** 2004 [35]	Retrospective (single-center)	10	8 uLMS, 1 EES, 1 ADNS	80% doxo + cisplatin, 20% doxo + MMC	ND	CC0: 90 CC2: 10	0	0	30	65	ND
**Baratti ** 2010 [16]	Retrospective (single-center)	11 of 37	11 uLMS	doxo + MMC or cisplatin	mean 14.7 (2–34)	Overall CC0: 76 CC0-1: 84	Overall 21.6	Overall 27	ND median uLMS 15 months	uLMS 40 (best results)	uLSM 29.5
**Sardi** 2017 [18]	Retrospective (multicenter)	36	29 uLMS, 3 EES, 3 ADNS, 1 other	22 doxo + cisplatin, 10 melphalanand 4 others: cisplatin/MMC	median 16 (2–39)	CC0-1: 94	21	2.8	LMS 39 (<20 at 2 years in others)	Overall 32 (LMS 41, Others < 29)	LMS 37
**Díaz-Montes** 2018 [36]	Retrospective (single-center)	26 (7 CRS + HIPEC, 5 no CRS; 14 CRS)	22 uLMS, 2EES, 2 ADNS	melphalan	ND	CRS: 79 CC0; Group CRS + HIPEC: 100 CC0	1 patient (20% Group CRS + HIPEC)	0	ND median group HIPEC 11.3 m; CRS 5.3 m	ND	CRS + HIPEC: 43.8; CRS: 35.9
**Düzgün** 2022 [37]	Retrospective (single-center)	8 of 22	5 uLMS, 3 EES	doxo + cisplatin	mean 12.8 (3–15)	Overall CC0: 73 CC0-1: 86	Overall 31.8	0	Overall 36	Overall 57	Overall 45.3
**Muñoz-Casares** 2024 [21]	Retrospective (single-center)	10 of 23	5 EES, 3 uLMS, 2 UUS	70% doxo + cisplatin, cisplatin, paclitaxel	median 17 (3–36)	Overall CC0: 87, CC0-1: 96	Overall 13	0	Overall 34.5 (US 34) (LG-EES 67)	Overall 64.6 (US 56) (LG-EES 100)	ND

Abbreviations: ADNS: adenosarcoma; CCS: completeness of cytoreduction score; CRS: cytoreductive surgery; DFS-5y: disease-free survival at 5 years; Doxo: doxorubicin; EES: endometrial stromal sarcoma; HIPEC: hyperthermic intraperitoneal chemotherapy; LG-EES: low grade endometrial stromal sarcoma; MMC: mitomycin C; ND: no determination; OS-5y: overall survival at 5 years; uLMS: uterine leiomyosarcoma; US: uterine sarcoma; UUS: undifferentiated uterine sarcoma.

**Table 4:** Clinical recommendations applicable in the systemic treatment of uterine peritoneal sarcomatosis.

**Clinical recommendations and levels of evidence in systemic treatment for advanced uterine sarcomas according to published Clinical Practice Guidelines [26]**
**High-grade uterine sarcoma**
√ In fit patients with uLMS (uterine leiomyosarcoma), the combination of doxorubicin plus trabectedin should be the preferred first-line chemotherapy, particularly when obtaining a response is relevant (I, A). Nevertheless, single-agent doxorubicin and doxorubicin/dacarbazine are valuable alternatives. √ In non-uLMS histologies, single-agent doxorubicin should be considered standard (I, B). However, anthracycline-based combinations (with ifosfamide or dacarbazine) are valuable options when obtaining a response is relevant. √ Trabectedin, gemcitabine combinations (preferably with dacarbazine), and pazopanib are second and further-line options depending on the first-line chemotherapy regimen received. √ Endocrine therapy with aromatase inhibitors may be a reasonable option for some patients with advanced ER/ PR-positive uLMS. √ NTRK fusion testing could be considered in uterine sarcomas. Patients with NTRK-positive tumors should be offered an NTRK inhibitor [III, A].
**Low-grade uterine sarcoma**
√ Treatment with aromatase inhibitors or progestins is recommended as first-line treatment. Due to their better toxicity profile, aromatase inhibitors are preferred as first-line hormonal therapy (III, A).

**Table 5:** Published series of patients with peritoneal sarcomatosis of retroperitoneal origin undergoing surgery using CRS-HIPEC.

Author and Year	Design Study	Patients (n)	Primary Tumor Histology	HIPEC	PCI	CCS (%)	Morbidity G 3,4 (%)	Mortality 30 d (%)	DFS-5y (%)	OS-5y (%)	OS Median (months)
**Berthet** 1999 [9]	Retrospective (single-center)	16 of 43 (30 PS)	22 LMS, 9 LPS, 4 FS, 4 DSRCT, 1 MPNST, 1 SFT	HIPEC with cisplatin (3 HIPEC, 13 HIPEC+ EPIC); 14 EPIC, 13 No PS	9 with <1334 with >13	CC0-1: 63	19	7	ND	Overall 39 (CC0-1)	Overall 20
**Rossi** 2004 [14]	Prospective (multicenter)	34 of 60	34 RPS (20 LPS, 6 UPS, 4 MPNST, 2 FS, 2 DSRCT); others	Doxo + cisplatin	mean 7.7 (2–21)	CC0: 68 CC0-1: 100	23	0	ND	Overall 38	Overall 36
**Baratti** 2010 [16]	Retrospective (single-center)	13 of 37	13 LPS; others	doxo + MMC or cisplatin	mean 14.7 (2–34)	CC0: 76 CC0-1: 84	21.6	2.7	17.8	Overall 24	Overall 26 (LPS 34)
**Randle** 2013 [43]	Retrospective (single-center)	7	2 SFT, 2 SCS, 1 LMS, 1 FS, 1 DSRCT	MMC ± MTX ± cisplatin	ND	CC0-1: 60	50	0	ND	43	21.6
**Sommariva** 2013 [44]	Retrospective (single-center)	8 of 15	3 LPS, 1 LMS, 1 UPS, 1 MPNST, 1 DSRCT, 1 SS; others	doxo + MMC or doxo + cisplatin	median 5.5 (2–15)	CC0: 93	ND	ND	17.4	29	27
**Abu-Zaid** 2016 [45]	Retrospective (single-center)	11	11 RPS (7 LPS, 4 no LPS)	6 doxo + cisplatin, 4 melphalan, 1 MMC	median 14 (3–29)	CC0: 64 CC0-1: 100	9	0	ND	ND	28.3
**Karamveri** 2019 [46]	Retrospective (single-center)	16 of 20	5 LPSDD, 5 RMS, 4 LMS, 2 LPSWD; others	doxo + cisplatin	mean 6 (2–24)	CC0: 86	20.7	0	ND	43	55
**Spiliotis** 2021 [19]	Retrospective (multicenter)	21	7 LPS, 6 LMS, 4 RMS, 4 FS	11 MMC, 7 doxo, 3 cisplatin	median 10 (3–20)	CC0: 52 CC0-1: 90	14.3	4.7	ND	ND	20.5
**Almasri** 2024 [20]	Retrospective (single-center)	29 (ND % RPS)	12 LPS, 7 LMS, 3 FS, 2 UPS, 5 others	ifosfamide iv + HIPEC (24 doxo + cisplatin, 5 doxo + MMC)	median 6 (3–12)	CC0: 52 CC0-1: 69	31	0	ND (35 at 2 years)	ND (73 at 2 years)	ND

Abbreviations: CCS: completeness of cytoreduction score; CRS: cytoreductive surgery; DFS-5y: disease-free survival at 5 years; Doxo: doxorubicin; DSRCT: desmoplastic small round cell tumor; EPIC: early postoperative intraperitoneal chemotherapy; FS: fibrosarcoma; HIPEC: hyperthermic intraperitoneal chemotherapy; LMS: leiomyosarcoma; LPS: liposarcoma; LPSDD: dedifferentiated liposarcoma; LPSWD: well-differentiated liposarcoma; MMC: mitomycin C; MPNST: malignant peripheral nerve sheath tumor; ND: no determination; OS-5y: overall survival at 5 years; RMS: rhabdomyosarcoma; RPS: retroperitoneal sarcoma; SCS: spindle cell sarcoma; SFT: solitary fibrous tumor; SS: synovial sarcoma; UPS: undifferentiated pleomorphic sarcoma.

**Table 6:** Clinical recommendations applicable in the systemic treatment of peritoneal sarcomatosis of retroperitoneal origin.

**Clinical recommendations and levels of evidence in systemic treatment for advanced retroperitoneal sarcomas** **according to published Clinical Practice Guidelines [42,47,48,49]**
√ In low-grade RPS, especially in asymptomatic patients, active surveillance may be a good option (IV, C). √ Anthracycline-based chemotherapy is the standard first-line treatment of advanced disease (II, A). Anthracycline-based combinations (II, A) can be evaluated in fit patients, when surgical salvage is the goal, and in patients in whom a dimensional response might improve symptoms. √ Several second-line and subsequent treatments are available for the treatment of patients after progression or in those who are ineligible for first-line, and the decision is based on histology, toxicity profile, and patient preference (IV, C). √ Although there is additional evidence of trabectedin activity in L-sarcomas (I, A), it can be considered in the treatment of all sarcoma subtypes (III, B). √ Pazopanib is indicated in the treatment of non-LPS (II, A). √ Eribulin is an alternative in the treatment of LPS (I, A). √ Gemcitabine combinations, preferably with DTIC, due to a better tolerability profile, are an especially useful alternative in LMS (II, B). √ High-dose ifosfamide is an option, particularly in synovial sarcoma (III, B). √ Inclusion in clinical trials for advanced disease patients is highly recommended (V, A). √ NGS and other -omics are needed to increase knowledge, select patients for clinical trials, and identify potential driven treatments (V, A).

**Table 7:** Published series of patients with peritoneal GISTosis undergoing surgery using CRS-HIPEC.

Author and Year	Design Study	Patients (n)	Primary Tumor Histology	HIPEC	PCI	CCS (%)	Morbidity G 3,4 (%)	Mortality 30 d (%)	DFS-5y (%)	OS-5y (%)	OS Median (months)
**Rossi** 2004 [14]	Prospective (multicenter)	14 of 60	14 GIST pre-TKI, 46 others	doxo + CDDP	mean 7.7 (2–21)	Overall CC0: 68 CC0-1: 100	Overall 23	0	ND	Overall 38	Overall 34
**Lim** 2007 [15]	Prospective Comparative Phase 1. Non-randomized	17 of 28	17 LMS/GIST pre-TKI, 11 others	19 HIPEC: CDDP 9 HIPEC: CDDP + MTX	ND	Overall CC0-1:95 (group CDDP) vs. 100 (CDDP + MTX)	16 (CDDP) vs. 44 (CDDP + MTX)	0	ND	ND	Overall 16.9 CDDP vs. 5.5 CDDP + MMC group
**Baratti** 2010 [16]	Retrospective (single-center)	8 of 37	8 GIST pre-TKI, 29 others	doxo + MMC or CDDP	mean 14.7 (2–34)	Overall CC0: 76 CC0-1: 84	Overall 21.6	Overall 2.7	Overall 17.8	Overall 24.3	Overall 26 (GIST 18)
**Baumgartner** 2013 [62]	Retrospective (single-center)	2 of 15	2 GIST, 13 others	NSD (Overall MMC 82%, CDDP 12%, doxo 6%)	ND	Overall CC0: 82 CC0-1: 100	Overall 24	0	ND	Overall >35	Overall 22.6 (GIST 23.9)
**Bryan** 2014 [63]	Retrospective (single-center)	16 (50% pre-TKI)	62.5% GIST small intestine, 31.3% GIST stomach	MMC ± MTX	ND	CC0-1: 72	Overall 33.3	5.6	ND	ND (at 3-years: 56)	41 (94 with TKI, 12 no TKI)
**Muñoz-Casares** 2024 [21]	Retrospective (single-center)	6 of 23	6 GIST, 17 others	doxo + CDDP	median17 (3-36)	Overall CC0: 87 CC0-1: 96	Overall 13	0	Overall 34.5 (GIST 33)	Overall 64.6 (GIST 80)	ND

Abbreviations: CCS: completeness of cytoreduction score; CDDP: cisplatin; CRS: cytoreductive surgery; DFS-5y: disease-free survival at 5 years; Doxo: doxorubicin; GIST: gastrointestinal stromal tumor; HIPEC: hyperthermic intraperitoneal chemotherapy; LMS: leiomyosarcoma; MMC: mitomycin C; MTX: mitoxantrone; ND: no determination; OS-5y: overall survival at 5 years; TKI: tyrosine kinase inhibitors.

**Table 8:** Clinical recommendations applicable in the systemic treatment of peritoneal GISTosis.

**Clinical recommendations and levels of evidence in systemic treatment for advanced or metastatic GIST according to published Clinical Practice Guidelines [53,66]**
**Imatinib for metastatic disease**
√ Genotype is mandatory for treating advanced/metastatic GIST patients (II, A). √ Imatinib 400 mg daily is the recommended dose in the first line (I, A). √ Imatinib 400 mg every 12 h is the recommended dose for GIST with *KIT* exon 9 mutation (II, A). √ It is unclear whether imatinib should be the first line in GIST KIT/PDGFRA WT (IV, C). √ Surgery is not recommended as a primary approach in the metastatic setting (I, A). √ Debulking surgery aiming an R0/R1 surgery can be considered in selected patients after initial response to imatinib (IV, B).
**Imatinib-resistant disease:**
√ Confirm adherence to treatment and rule out drug interactions at the time of progression to any TKI (III, B). √ After the failure of imatinib, the standard second-line treatment is sunitinib 50 mg daily 4/2 (I, A) or 37.5 mg continuously (III, C). √ Before sunitinib, imatinib dose escalation to 400 mg twice daily can be considered, particularly in patients with *KIT* exon 9-mutant GIST (III, B). √ Standard third- and fourth-line treatments are, respectively, regorafenib 160 mg daily 3/1 (I, A) and ripretinib 150 mg once daily (I, A). √ Avapritinib 300 mg daily is the only effective treatment available for PDGFRA D842V-mutant GIST and it should be introduced, if possible, as the first line (III, A). √ Maintenance of TKI pressure improves outcomes and it is advised while an alternative therapeutic option is unavailable (III, B). √ Surgery of unifocal/limited progression can be considered after discussion in a multidisciplinary tumor board (IV, C).

**Table 9:** Published series of patients with peritoneal sarcomatosis originating from non-GIST visceral sarcomas (DSRCT, RMS, EIMS) undergoing surgery using CRS-HIPEC.

Author and Year	Design Study	Patients (n)	Primary Tumor Histology	HIPEC	PCI	CCS (%)	Morbidity G 3,4 (%)	Mortality 30 d (%)	DFS-5y (%)	OS-5y (%)	OS Median (months)
**Hayes-Jordan** 2015 [17]	Retrospective (single-center)	28 of 50	21 DSRCT and 7 RMS; 22 others	CDDP	median 16	CC0: 95 CC0-1: 100	28	0	ND	DSRCT 30	DSRCT 31.4 (better results than RMS)
**Honoré** 2017 [78]	Retrospective (multicenter)	9 of 48 (only these 9 with HIPEC; 2 with EPIC)	9 DSRCT	CDDP + MMC or CDDP or oxaliplatin or CPT-11 + oxaliplatin	9 (2–27)	CC0-1: 100	HIPEC/EPIC Group 40 vs. Rest of groups 10	0	HIPEC/EPIC Group 0 vs. Rest of groups 14	HIPEC/EPIC Group 0 vs. Rest of groups 22	ND
**Hayes-Jordan ** 2018 [77]	Prospective Phase 2. Non-randomized	16 of 20	14 DSRCT and 2 RMS; 4 others	CDDP	median 15	CC0-1: 100	40	0	ND median DSRCT 14.8; others 13.9)	ND (83 at 3 years)	DSRCT 44.3 (better results than others 12.5)
**Scalabre** 2018 [79]	Retrospective (multicenter)	7 of 22	7 DSRCT; 15 others	ND	16 (4–26)	CC0: 73 CC0-1: 91	64	0	Mesoth > 60; rest of tumors 30	Mesoth 100; rest of tumors 50	Overall 57.5 (DSRCT 16.5)
**Gesche** 2019 [80]	Cases report	6	6 RMS (Embryonic RMS)	4 doxo + CDDP; 2 CDDP	median 5.5 (4-21)	CC0: 100	0	0	ND	ND	median follow-up 12 months (7–41): all alive
**Stiles** 2020 [81]	Retrospective (single-center)	9	9 DSRCT (6 with PS)	80% CDDP; melphalan or MMC	16 (5–20)	CC0: 50 CC0-1: 90	40	50	ND (at 3-years: 13)	ND (at 3-years: 55)	36 (CC0 45) (PCI < 16 best OS)
**Klingler** 2023 [2]	Retrospective (single-center)	4 DSRCT of 19 (only these 4 with HIPEC)	4 DSRCT; 15 others	CDDP	ND	CC0: 47	31.6	0	ND	40.2 in radical surgery vs. 13 in non-optimal	30 (DSRCT 17)
**Zhu** 2023 [82]	Cases report	8 of 19	7 RMS and 1 EIMS; 11 others	11 doxo + ifosfamide 5 doxo + CDDP; 3 CDDP	median 5 (2-21)	CC0-1: 100	10	0	ND median 12 months (1–31)	ND	14 patients alive with median follow-up of 12.5 months (1.5–31)

Abbreviations: CCS: completeness of cytoreduction score; CDDP: cisplatin; CPT-11: irinotecan; CRS: cytoreductive surgery; DFS-5y: disease-free survival at 5 years; Doxo: doxorubicin; DSRCT: desmoplastic small round cell tumor; EIMS: epithelioid inflammatory myofibroblastic sarcoma; EPIC: early postoperative Intraperitoneal chemotherapy; HIPEC: hyperthermic intraperitoneal chemotherapy; MMC: mitomycin C; Mesoth: mesothelioma; ND: no determination; OS-5y: overall survival at 5 years; PCI: Peritoneal Cancer Index; PS: peritoneal sarcomatosis; RMS: rhabdomyosarcoma.

**Table 11:** Recommendations and Levels of Evidence.

Recommendations and Levels of Evidence	Voters (n)	Answer Yes (n)	Answer No (n)	Consensus
**1. GENERAL PATIENT MANAGEMENT**				
(1.1) It should be performed in a high-volume sarcoma center that has a committee made up of a multidisciplinary team and experienced peritoneal and retroperitoneal oncological surgeons (III, A)	46	46	0	100% unanimous
**2. DIAGNOSTIC EVALUATION**				
(2.1) CT thorax–abdomen–pelvis c/c will help us evaluate disease, extension, and biopsy options. Given its contribution and greater availability, it should be the initial imaging test (IV, A)	46	45	1	98% strong
(2.2) MRI allows obtaining multiphasic images with contrast, enhanced in diffusion, which facilitates the detection of disease in difficult sites such as the mesentery, serosa of the small intestine and pelvis. It should be considered a complementary option to CT (IV, A)	47	45	2	96% strong
(2.3) PET/CT will be useful to confirm doubtful disease or rule out lymph node disease and distant metastases. It should be considered complementary to CT and MRI (IV, A)	48	44	4	92% strong
(2.4) It is recommended that the biopsy be performed using Core Needle Biopsy (IV, A)	44	42	2	95% strong
(2.5) We must know the histological subtype assessed by an expert pathologist and, prior to the initial therapeutic decision, by an experienced multidisciplinary team (IV, A)	47	47	0	100% unanimous
**3. INDUCTION TREATMENT/INITIAL TREATMENT**				
(3.1) In patients with metastatic sarcoma, especially with sensitive and high-grade histologies, systemic treatment is the first choice (IV, B). Its response and non-progression will allow evaluation of the options for radical cytoreductive surgery (CRS) in peritoneal sarcomatosis (V, B)	44	42	2	95% strong
(3.2) In peritoneal gistosis, it is mandatory to know the genotype to adapt the induction treatment (II, A). Imatinib will be the standard first-line treatment, except GIST without KIT/PDGFRA mutations or with PDGFRA exon 18 D842V mutation (I, A)	44	44	0	100% unanimous
(3.3) In peritoneal gistosis with failure of first-line TKI (Imatinib), it should be treated with successive lines (sunitinib, regorafenib, ripretinib) until response is achieved, as a prior step to assessing possible CRS (I, A)	45	39	6	87% weak
(3.4) In high-grade uterine peritoneal sarcomatosis type LMS, doxorubicin plus trabectedin (I, A) or doxorubicin plus dacarbazine (III, B) are currently the preferred first-line induction treatments. In potentially chemosensitive non-LMS histologies, anthracyclines and ifosfamide are the treatment choice to try to achieve surgical rescue (II, A)	43	41	2	95% strong
(3.5) In low-grade uterine peritoneal sarcomatosis, induction treatment using hormonal therapy with aromatase inhibitors is recommended as the first line (III, A)	43	40	3	93% strong
(3.6) In peritoneal sarcomatosis originating from high-grade retroperitoneal sarcomas, anthracycline-based combinations represent the induction treatment when the objective is surgical rescue (II, A). In the second line, there are other options to achieve a response, condition prior to CRS: trabectedin and eribulin in LPS (I, A), pazopanib in non-LPS (II, A), combinations of gemcitabine with dacarbazine in LMS (II, B)	42	38	4	90% strong
(3.7) In peritoneal sarcomatosis originating from low-grade retroperitoneal sarcomas, such as well-differentiated LPS, we do not have an effective induction treatment, so we should consider CRS with the aim of achieving complete cytoreduction (IV, B)	44	44	0	100% unanimous
(3.8) In peritoneal sarcomatosis due to desmoplastic small round cell tumor, induction chemotherapy based on combinations of alkylating agents, similar to Ewing sarcomas, followed by aggressive cytoreductive surgery, represents the standard treatment (IV, B)	44	43	1	98% strong
(3.9) In peritoneal sarcomatosis due to epithelioid inflammatory myofibroblastic sarcoma with ALK mutations, targeted therapy with ALK inhibitors (crizotinib) will be the standard first-line treatment (II, A) and its possible combination with complete cytoreductive surgery, after confirming response, would represent the choice strategy (IV, A)	44	44	0	100% unanimous
(3.10) In peritoneal sarcomatosis due to rhabdomyosarcoma, the initial chemotherapy regimens of choice for its most common variants, embryonal and alveolar, include ifosfamide, vincristine, actinomycin D, doxorubicin, cyclophosphamide, and vinorelbine (IV, A). Subsequent CRS with complete cytoreduction is the best option (IV, B)	42	41	1	98% strong
**4. RADICAL CYTOREDUCTIVE SURGERY (CRS)**				
(4.1) CRS with peritonectomy procedures, following the Sugarbaker principles, represents the best surgical approach to try to achieve complete macroscopic cytoreduction in peritoneal sarcomatosis (II, B)	42	40	2	95% strong
(4.2) Incomplete cytoreduction confers no survival benefit and may lead to significant morbidity (IV, B)	46	39	7	85% Weak
(4.3) In peritoneal GISTosis, CRS can be considered with the aim of achieving complete cytoreduction in selected patients, after 6–12 months of induction treatment, with response to imatinib (IV, B) or even to other lines of TKI (IV, C)	44	42	2	95% Strong
(4.4) In peritoneal GISTosis, CRS can be considered with the objective of achieving complete cytoreduction, in selected patients with partial clinical response and oligoprogression (limited unifocal progression) to TKI treatment (IV, C)	43	39	4	91% Strong
(4.5) In peritoneal GISTosis with progression to different TKI lines, radical cytoreductive surgery should be avoided (IV, C)	43	35	8	81% Weak
(4.6) In non-GIST peritoneal sarcomatosis, with response to induction treatment, they will be candidates for CRS if there are complete cytoreduction options (IV, B)	43	42	1	98% Strong
(4.7) Patients with high-grade peritoneal sarcomatosis and very high PCI with involvement of all abdominal compartments, despite response to induction treatment, should be evaluated in a Committee with a multidisciplinary team in reference to possible CRS versus other therapeutic options (V, C)	44	37	7	84% Weak
(4.8) In a patient with peritoneal sarcomatosis and distant metastasis (liver or lung) who has responded to previous systemic treatment, we should not rule out CRS if there are options for complete cytoreduction of both (V, C)	45	38	7	84% Weak
**5. HYPERTHERMIC INTRAOPERATIVE CHEMOTHERAPY (HIPEC)**				
(5.1) HIPEC is a complement used in CRS after CC0, with little evidence in peritoneal sarcomatosis, with doxorubicin+cisplatin being the most frequently used scheme (V, C)	40	39	1	98% Strong
(5.2) In peritoneal GISTosis, HIPEC lacks a rational basis as GIST is not sensitive to conventional chemotherapy, so it should not be used, except in high-volume referral centers with experience in these procedures and always under clinical investigation (V, C)	44	42	2	95% Strong
(5.3) In non-GIST peritoneal sarcomatosis, the evidence of the role of HIPEC after CRS is unknown, so it is recommended that its administration is carried out exclusively in those patients with a response to induction chemotherapy in whom complete cytoreduction is achieved, in referring Centers and under clinical investigation (V, C)	43	38	5	88% Weak
**6. POSTOPERATIVE ADJUVANT TREATMENT**				
(6.1) The Committee formed by the multidisciplinary team will assess the different histological subtypes in each particular situation and possible adjuvant or directed therapeutic options (V, C)	44	44	0	100% unanimous
(6.2) In patients with peritoneal GISTosis, with response to induction treatment with Imatinib and subsequent CRS with complete cytoreduction, treatment with Imatinib will be maintained until disease progression or unacceptable toxicity (I, A)	43	41	2	95% strong
**7. FOLLOW-UP**				
(7.1) Surveillance with imaging every 3–6 months is justified after complete cytoreductive surgery for peritoneal sarcomatosis, as many patients will develop recurrent metastases and some will be candidates for additional local or systemic treatment, which must be decided within the Committee formed by the multidisciplinary team (IV, A)	43	41	2	95% strong
(7.2) CT thorax–abdomen–pelvis c/c, given its contribution as an imaging diagnosis and its usual greater availability, is the standard test for the follow-up of patients treated for peritoneal sarcomatosis (IV, A)	44	44	0	100% unanimous
(7.3) In the event of abdominal recurrence after previous CRS with complete cytoreduction, a new CRS will be recommended as long as it presents a response to systemic treatment and/or has the possibility of new complete cytoreduction (V, C)	44	39	5	89% weak
**8. FINAL RECOMMENDATION**				
(8.1) In patients with advanced metastatic sarcoma such as peritoneal sarcomatosis, inclusion in clinical trials is recommended (V, A)	46	44	2	96% strong
